# Human Plasma Metabolomics for Biomarker Discovery: Targeting the Molecular Subtypes in Breast Cancer

**DOI:** 10.3390/cancers13010147

**Published:** 2021-01-05

**Authors:** Leticia Díaz-Beltrán, Carmen González-Olmedo, Natalia Luque-Caro, Caridad Díaz, Ariadna Martín-Blázquez, Mónica Fernández-Navarro, Ana Laura Ortega-Granados, Fernando Gálvez-Montosa, Francisca Vicente, José Pérez del Palacio, Pedro Sánchez-Rovira

**Affiliations:** 1 Medical Oncology Unit, University Hospital of Jaén, 23007 Jaén, Andalucía, Spain; leticia.diaz@juntadeandalucia.es (L.D.-B.); carmengo.92@gmail.com (C.G.-O.); natalia.luque.sspa@juntadeandalucia.es (N.L.-C.); musikafn@gmail.com (M.F.-N.); analaura.ortega.sspa@juntadeandalucia.es (A.L.O.-G.); fernando.galvez.sspa@juntadeandalucia.es (F.G.-M.); 2 Fundación MEDINA, Centro de Excelencia en Investigación de Medicamentos Innovadores en Andalucía, 18016 Granada, Andalucía, Spain; ariadna.martin@medinaandalucia.es (A.M.-B.); francisca.vicente@medinaandalucia.es (F.V.); jose.perezdelpalacio@medinaandalucia.es (J.P.d.P.)

**Keywords:** human plasma metabolomics, breast cancer, molecular subtypes, metabolic profiling, personalized medicine

## Abstract

**Simple Summary:**

Breast cancer is the leading cause of female cancer-related deaths worldwide. New technologies with enhanced sensitivity and specificity for early diagnosis and tailored monitoring are in critical demand. Thus, metabolomics appears to be a growing tool in order to detect molecular differences between distinct groups. In this case, an untargeted analytical approach was used to identify metabolomics differences between molecular subtypes of breast cancer in comparison with healthy matched controls. Footprints for each breast cancer subtype provided diagnostic capacities with an area under the receiver-operating characteristic curve above 0.85, which suggests that our results may represent a major advance towards the improvement of personalized medicine and precise targeted therapies for the various breast cancer phenotypes. To validate these molecular profiling as potential therapeutic strategies for the different breast cancer subtypes, further analysis and larger cohorts would be necessary in the near future.

**Abstract:**

Purpose: The aim of this study is to identify differential metabolomic signatures in plasma samples of distinct subtypes of breast cancer patients that could be used in clinical practice as diagnostic biomarkers for these molecular phenotypes and to provide a more individualized and accurate therapeutic procedure. Methods: Untargeted LC-HRMS metabolomics approach in positive and negative electrospray ionization mode was used to analyze plasma samples from LA, LB, HER2+ and TN breast cancer patients and healthy controls in order to determine specific metabolomic profiles through univariate and multivariate statistical data analysis. Results: We tentatively identified altered metabolites displaying concentration variations among the four breast cancer molecular subtypes. We found a biomarker panel of 5 candidates in LA, 7 in LB, 5 in HER2 and 3 in TN that were able to discriminate each breast cancer subtype with a false discovery range corrected *p*-value < 0.05 and a fold-change cutoff value > 1.3. The model clinical value was evaluated with the AUROC, providing diagnostic capacities above 0.85. Conclusion: Our study identifies metabolic profiling differences in molecular phenotypes of breast cancer. This may represent a key step towards therapy improvement in personalized medicine and prioritization of tailored therapeutic intervention strategies.

## 1. Introduction

Breast cancer (BC) is currently the most common malignancy in women, both in developed and less developed countries, and the leading cause of cancer-related deaths among women worldwide, with a high incidence rate [1,2]. Every breast cancer subtype is characterized by intrinsic molecular features and metastatic lesions, and their natural heterogeneity leads to a high diversity in prognosis and clinical responses to available medical treatments, even for patients with similar diagnosis, histology and stage of disease [3,4,5,6,7,8,9]. 

Therefore, determining the molecular subtypes of breast cancer becomes crucial for personalized treatment. In fact, there is evidence reporting that patients receiving molecular-matched therapy have an increased overall response rate, longer period of time to treatment failure and a longer survival rate in comparison to patients with non-matched therapy [3,9]. Successive biopsy procedures and subsequent histopathological analysis are normally used to study molecular and genetic information from tumor cells in order to diagnose and classify BC into a subtype. This analytical technique is invasive and time consuming [3]. Thus, non-invasive, fast, sensible and precise analytical methods for distinction of different BC subtypes are in critical demand [10,11]. 

In this sense, metabolomics has quickly arisen as a novel approach in the cancer biomarker field to overcome the current limitations of standard diagnostic techniques [12]. This expanding research area provides a dynamic portrait of an individual overall metabolic status, assessing the final products of the myriad of intrinsic molecular processes and intercellular pathways that may be altered in response to genetic, pathological and/or environmental factors [3,13]. Hence, the end products of the diverse biological processes known as metabolites can be analyzed from high-throughput screening technologies such as nuclear magnetic resonance (NMR) and mass spectrometry (MS) enabling the discovery of altered pathways that may give us new insights into dysregulated metabolism in tumor development and progression. Therefore, the altered metabolites reflecting these pathophysiological changes might be considered as potential new therapeutic targets for breast cancer diagnosis, prognosis, early recurrence and drug efficacy [14,15,16]. 

Several studies have already been conducted to explore the possibility of using metabolite panels as biomarkers for early diagnosis, tumor characterization and clinical outcome prediction [3,14,15,16,17,18,19,20]. Human body fluids such as saliva, urine, serum and plasma have been re-discovered as a great source of potential biological markers, and hence analyzed in the search of a metabolic profile that may be representative of systemic metabolic dysregulation in breast cancer patients [19,20,21,22,23]. However, up to today, efforts on proving highly accurate markers or proven targets for tailored therapeutic treatments have not yet delivered the expected results [24,25,26,27,28] due to the high heterogeneity displayed by breast cancer, from histology to prognosis, early recurrence, risk of metastatic progression or response to treatment and survival rates [29].

With this aim in view, we explore whether metabolomics is able to provide an accurate pathological diagnosis, phenotypic classification and a tailored follow-up of individuals with this malignancy. A high-throughput untargeted metabolic approach was used to identify the capacity of different metabolic profiles to predict various BC subtypes. Based on a liquid chromatography-mass spectrometry (HPLC/Q-TOF 5600) platform-based metabolomics study, we propose and test the notion that a differential metabolic signature representative of the distinct breast cancer subtypes exists, and it can be ultimately detected in plasma of individuals with this disease. 

## 2. Results

### 2.1. Patients’ Characteristics

To avoid the effect of potential confounding variables like age and Body Mass Index (BMI), the homogeneity of BC group and its corresponding HC subjects was evaluated. Normality’s distribution was checked with a Shapiro-Wilk normality test and the equality of variances of both study groups was studied with the Levene´s test when corresponded. Finally, the appropriated *t* test was applied without significant differences observed in any case.

### 2.2. LC-HRMS Analysis

**Figure 1:**
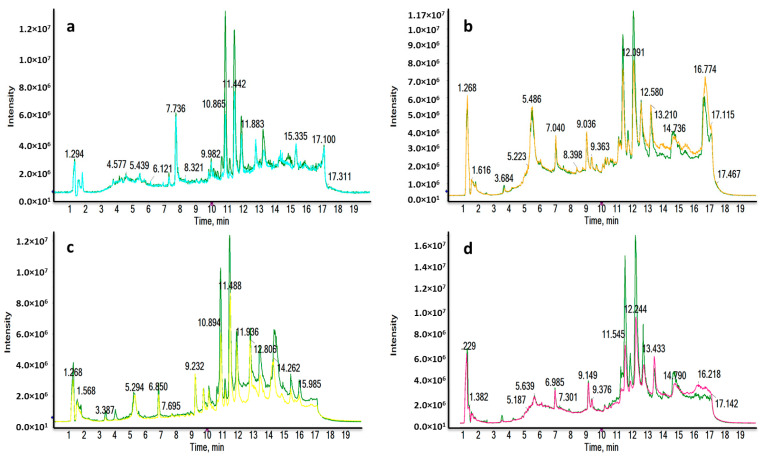
Representative RPLC-ESI+-HRMS TICs of a LA_BC (light blue) (**a**), HER2_BC (orange) (**b**), TN_BC (yellow) (**c**) and LB_BC (pink) (**d**) sample compared to a HC sample (green). Remarkable differences were observed between BC and HC samples.

Four different liquid chromatography-high resolution mass spectrometry (LC-HRMS) analyses were carried out for each ionization mode, in order to determine the molecular differences between the major subtypes of breast cancer (luminal A (LA), luminal B (LB), triple negative (TN) and human growth factor receptor 2 positive (HER2) and the healthy control (HC) groups. The reverse phase (RP) column is recommended for the separation of medium-polar metabolites (such as phospholipids, lysophospholipids or steroids) and non-polar metabolites. Total ion chromatograms (TICs) in positive electrospray ionization mode (ESI+) are shown in Figure 1, where clear differences are observed between BC subtypes and HC groups corresponding to the most significant discriminatory features detected: very polar metabolites eluted in the first 3 min (Figure 1a,c); medium-polar metabolites were found to elute from 8.5 to 12.5 min (Figure 1a,b,d); non-polar metabolites were not found in our work to be discriminatory after all the statistical analysis.

### 2.3. Chemometric Analysis

**Figure 2:**
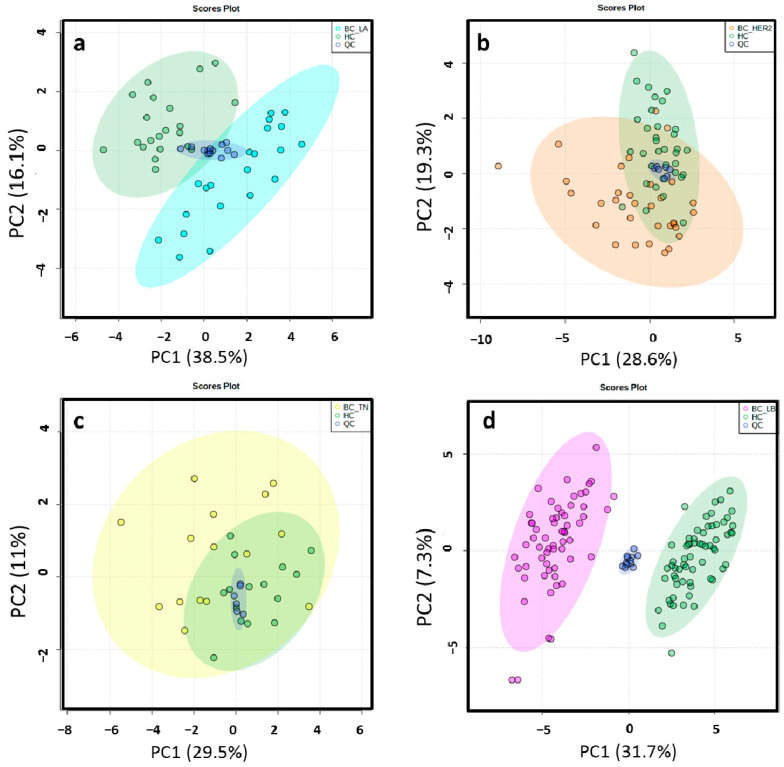
2D score plots of the unsupervised PCA of HC group (green) and LA_BC (light blue) (**a**), HER2_BC (orange) (**b**), TN_BC (yellow) (**c**) and LB_BC (pink) (**d**) patients by ESI− showed that the separation observed between the groups was due to biological reasons according to the close clustering of the QC samples (dark blue).

**Figure 3:**
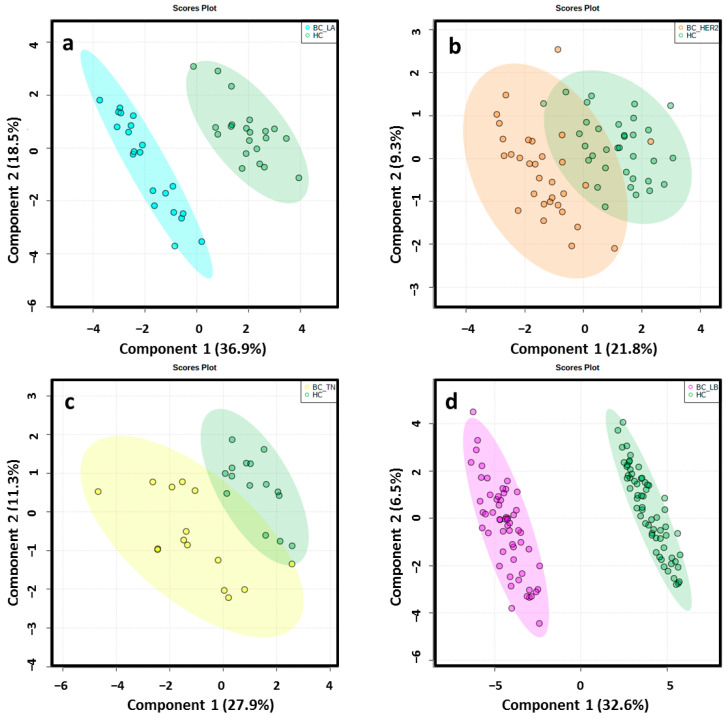
2D score plots of the supervised PLS-DA of HC group (green) and LA_BC (light blue) (**a**), HER2_BC (orange) (**b**), TN_BC (yellow) (**c**) and LB_BC (pink) (**d**) patients by ESI−determined a notably separation between BC molecular subtypes and matched controls.

Different data matrices were obtained depending on the ionization mode and the set of BC molecular subtype analyzed. Retention time (RT) windows and mass tolerances were determined for each analyzed set based on the data of selected chromatographic peaks. After monoisotopic selection, contaminants were removed based on the organic solvent (OS) filtration and several features presented in the quality control (QC) samples were excluded for unacceptable variability (relative standard deviation > 30%). Remaining variables were evaluated by multivariate statistical analysis (Table S1). The close clustering of the QC samples in Figure 2 indicates that the separation observed between the corresponding study groups was mainly due to biological reasons in ESI−. The authors found that PC1 and PC2 explained 54.6%, 47.9%, 40.5% and 39% of the total of variance in LA, HER2, TN, LB in the ESI− mode analysis, respectively. The variance obtained with PC1 and PC2 was 42.5%, 40.5%, 44.8% and 43% in LA, HER2, TN, LB in the ESI+ mode analysis, respectively. Unsupervised principal component analysis (PCA) score plots obtained by ESI+ are shown in Figure S1. Score plots of the partial least squares-discriminant analysis (PLS-DA) models illustrated a marked separation between the HC group and BC molecular subtypes by both ESI modes (Figure 3 and Figure S2); the “goodness” of the PLS-DA model, measured by R^2^ and Q^2^, showed that no over-fitting was observed and, consequently, these models are acknowledged for successful discernment between HC patients and the LA, LB, TN and HER2 BC molecular subtypes [30] (Table S1). Signals with false discovery range (FDR) corrected *p*-values < 0.05 were selected as altered metabolites; those with a fold-change (FC) value of at least 1.3 between the study groups were selected as potential biomarkers (BM) to identify.

### 2.4. Differential Metabolomic Profiling

A tentative identification of the final candidates was achieved as it was previously reported by the Schymansky classification. All identified metabolites were classified at level 1 and 2, therefore, their identities or probable structures are confirmed [31,32]. Hence, 5 metabolites were defined for the LA phenotype, 7 for LB, 5 for HER2 and 3 for TN (Table 1). The rest of metabolites (Table S2) met the criteria established for potential biomarkers of BC, although they could not be identified due to their MS/MS pattern, which did not match any of the queries of the compound databases searched (Metlin, Human Metabolome Database, Lipid Maps, PubChem, MassBank and NIST) or commercial standards used. This is likely to happen since the major part of the identity queries belonged to a similar molecular family whose virtual MS/MS spectra differences needed to be clarified, or because some of the signals have not been discovered yet. 

**Figure 4:**
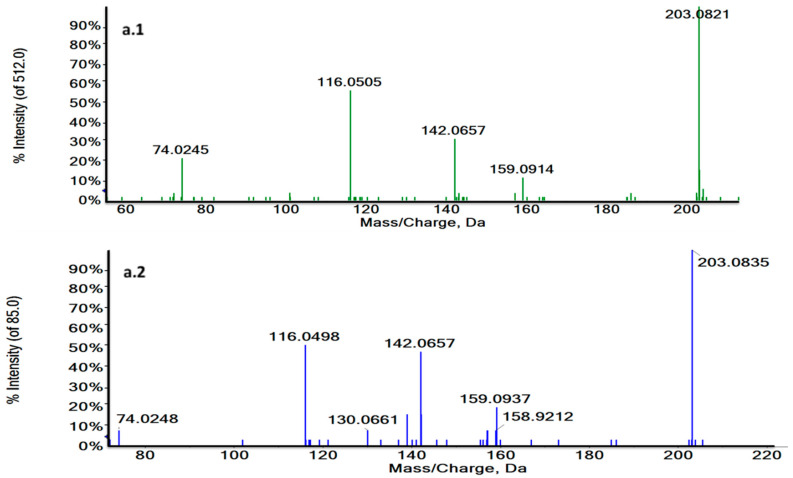

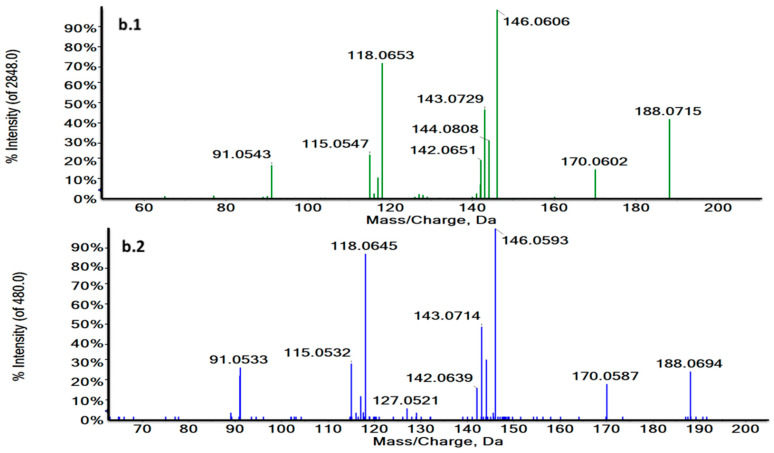
Characteristic MS/MS spectra of *m/z* 188.0707 in a biological sample (green) (**a.1**) and the L-Tryptophan standard at 3.73 min in ESI + (blue) (**a.2**) and *m/z* 203.0824 at 1.27 min in a biological sample (green) (**b.1**) and the L-Tryptophan standard in ESI− (blue) (**b.2**). MS/MS spectra revealed the characteristic fragmentation pattern of L-Tryptophan both in ESI+ and ESI−.

Thus, RT and MS/MS spectra of L-Tryptophan and Glycoursodeoxycholic acid (GUDCA) could be compared with their commercial standards under the same analytical conditions (Figure 4a,b and Figure S3). The experimental pattern of these metabolites matched with their standards so that the tentative identity could be confirmed.

### 2.5. Biomarker Evaluation and Model Creation

**Figure 5:**
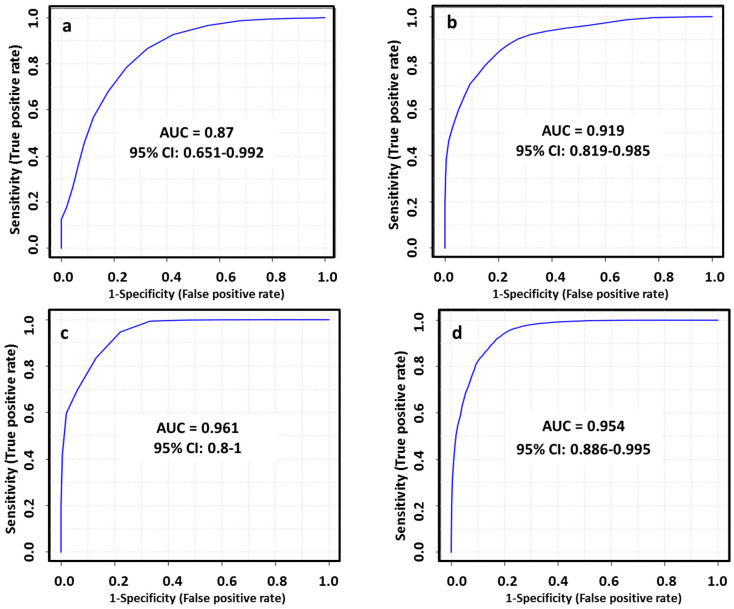
ROC curves for combined biomarkers model in LA_BC (**a**), HER2_BC (**b**), TN_BC (**c**) and LB_BC (**d**) by ESI+ and ESI−; 100 cross-validations were performed, and the results were averaged to generate the plot.

The diagnostic ability of the final tentatively identified candidates was evaluated with a multivariate receiver-operating characteristic (ROC) analysis. In this regard, we applied a PLS-DA model to combine our set of biomarkers to obtain the area under curve (AUC), which is a measure of how well a parameter can distinguish between two diagnostic groups. The AUC values obtained for each set of metabolites (Table 1) to discriminate between healthy patients and subtypes of breast cancer were 0.870, 0.919, 0.961 and 0.954 in LA, HER2+, TN and LB respectively. The performance of this biomarker model was evaluated using a balanced Monte Carlo cross-validation procedure. Although the model might improve when adding more of the potential biomarkers proposed in our work (Table S2), these features did not have a reliable structure ID since they could be only identified by their *m/z* and RT. Therefore, we preferred to use those metabolites based on the FDR corrected *p* value < 0.05, FC value > 1.3 and a tentative identification with a level classification of 1 or 2 by Schymansky (Table 1). The outcomes obtained for diagnostic potential of the selected biomarkers are summarized in Figure 5 and Table 2.

MetaboAnalyst 4.0 Web Server software (Wishart Research Group at the University of Alberta, Alberta, Canada) provided an average of predicted class probabilities of each sample in the 100 cross-validations. Confusion matrix in LA_BC revealed 14 BC and 16 HC samples correctly classified. Concurrently, 26 HER2_BC samples were correctly classified, whereas 28 samples were correctly distributed in the HC group. In TN_BC samples 13 BC and 11 HC samples were correctly classified; while 50 BC and 54 HC samples were properly assigned in LB_BC molecular subtype.

### 2.6. Pathway Analysis

We have found a set of biomarkers, which were able to discriminate each breast cancer subtype significantly. These first funding to distinguish at molecular level using untargeted metabolomics may improve the treatment of breast cancer and move towards to the priority of personalized medicine and customized therapeutic intervention strategies.

According to the deregulated metabolites tentatively identified in each BC molecular subtype by ESI+ and ESI−, we determined the major altered pathways implicated in the four different subtypes. The outcomes were obtained by analyzing results in ESI+ and ESI−, differentiating by phenotypes. Thus, pathway analysis revealed that porphyrin and chlorophyll metabolism, glycerophospholipid metabolism, tryptophan metabolism and aminoacyl-tRNA biosynthesis appeared to be altered (Table S3). Statistically significant pathways (*p* < 0.05) are shown in Table 3.

Pathway Analysis using MetaboAnalyst 4.0 Web Server software revealed two statistically significant dysregulated pathways (*p* value < 0.05) in breast cancer molecular subtypes.

## 3. Discussion

The advent of the –omics techniques is substantially accelerating predictive, preventing and personalized medicine. Next-generation sequencing (NGS), genomics and transcriptomics provide a better understanding of the genomic architecture of cancer and allow the discovery of differentially expressed genes that drive and maintain tumorigenesis. Genomic profiling has yielded potential biomarkers clinically relevant for early diagnosis of breast cancer, but these analytical platforms have some disadvantages, like shorter read lengths that challenges genome alignment and assemble, how to navigate through mega-datasets and, additionally, their cost is still high in comparison with other techniques. In contrast with the gene panels discovered by other techniques, metabolites are closer to the phenotype of the organism than genes and proteins, so the metabolome can be a point of convergence for genetic variation influencing complex traits, and can efficiently elucidate the mechanisms underlying phenotypic variation. Thus, metabolomics profiling is considered as a relatively more rapid, accurate and non-invasive method to discover diagnostic and prognostic biomarkers. In this work, we applied an untargeted high-throughput metabolomics approach to compare the plasma metabolic profiling changes associated with the distinct BC molecular subtypes (LA, LB, TN and HER2) versus healthy controls. By using RPLC-HRMS in ESI+ and ESI− modes, we were able to detect statistically significant differences in certain metabolites with high diagnostic capacity in the four different BC phenotypes, which are involved in relevant biological cancer-related pathways such as: glycerophospholipid metabolism, porphyrin and chlorophyll metabolism, tryptophan metabolism and aminoacyl-tRNA biosynthesis.

Otto Warbug described in great detail how cancer cells increase their glucose consumption as a fuel source to support the anabolic processes that promote their uncontrolled proliferation. Not only have Warburg’s findings been confirmed, but other catabolic pathways have demonstrated their fundamental role in cancer progression [33,34]. Our findings go in accordance with the essential necessity of upregulating the energy supply in breast cancer cell growth and proliferation. Interestingly, a significant decreased concentration of L-Tryptophan (Trp) was observed in plasma of LA, TN and HER2 molecular subtypes of BC in comparison with healthy controls (FDR corrected *p* value < 0.05, FC < 0.6). Decreased tryptophan in plasma and serum of BC patients has also been reported in several studies [35,36,37,38]. Although the role of Trp catabolism in tumor proliferation is still unclear, it has been discovered to indirectly promote the degradation of the extracellular matrix and invasion on cancer cells [39]. Two main enzymes catalyze tryptophan into metabolites of the kynurenine (Kyn) pathway: tryptophan-degrading dioxygenases indoleamine-2,3-dioxygenase (IDO1) and tryptophan-2,3-dioxygenase (TDO2) [40,41]. Kyn activates the aryl hydrocarbon receptor (AhR) which contributes to cancer immune escape since it promotes an immunosuppressive tumor microenvironment by an increase of IL-10, Treg cells and suppressing immune activation cells [42]. Therefore, in cancer with an overexpression of IDO1/TDO2, increased Trp catabolism could lead to the depletion of its serum concentration and the accumulation of Kyn metabolites, which enhanced cancer scenario [43,44,45,46]. Nevertheless, up to date there are no IDO1/TDO2 inhibitors currently approved by the US Food and Drug Administration. The most recent clinical trial publishing the effect of an IDO1/TDO2 inhibitor, Indoximod (D-1MT/NLG-8189), did not show a clinical benefit in metastatic BC patients when combined with taxane chemotherapy [47]. In fact, a lot more research is needed in order to warrant the efficacy of these inhibitors in clinical practice [48].

The reprogramming of lipid metabolism is a hallmark of many cancers, including breast cancer. Several lipoids were identified to be differentially altered in LA, LB, TN and HER2 molecular subtypes when comparing with healthy controls, which emphasize the importance of investigating the lipid metabolism differences in breast cancer. Phospholipids are a main component of cell membranes, they play a major role in cell signaling and cycle regulation and are a source of fatty acids (FA) which oxidative metabolism and ATP production is critical, not only in normal cells but also in cancer function [49]. In particular, a decreased plasma concentration of phosphoethanolamines (LysoPE (16:0), (18:1), (18:2) FDR < 0.05 and fold change < 0.6) and phosphocholines (LysoPC (14:0), (16:0), (20:3) FDR < 0.05 and fold change < 0.7) was observed. Our findings are in line with the already suggested distinction in membrane dynamics across molecular subtypes of breast cancer, where the acyl-chain constituents of PC and PE is remodeled by the action of phospholipases and lysohpospholipid acyltransferases with the delivery of fatty acid molecules for structural, signaling, and energy-producing purposes of breast cancer cells [50]. However, in accordance with other studies, breast cancer cells adapt to metabolic stress under given experimental conditions (glutamine deprivation or serum deficiency), by changing PE and DAG homeostasis. In both cases, an accumulation of phosphoethanolamine (PEtn) was observed in breast cancer cells with reduced expression of PCYT2, suggesting tumor progression in response to glutamine deprivation [51,52]. Moreover, in conformity with a recent prospective study where 1624 first primary incident invasive breast cancer cases were compared by their molecular phenotypes with 1624 matched controls, a phosphatidylcholine (LysoPC (20:3)) was found to have a negative association with risk of breast cancer as we found in our analysis [53]. These biomarkers might open the possibility of identifying an early poor prognosis as well as detecting residual disease after neoadjuvant treatment (NAT).

Furthermore, only two non-related metabolites were found to be differently expressed under our experimental conditions in luminal A, luminal B and HER2 molecular subtypes: biliverdin and glycoursodeoxycholic acid. High levels of biliverdin (FDR < 0.05 and FC > 1.5) were detected in plasma of luminal B and HER2 cancer patients. Although both biliverdin (BV) and its catabolite bilirubin (BR) are non-toxic molecules that, under most conditions, act as anti-oxidants by scavenging or neutralizing reactive oxygen species (ROS) [54], they are also endogenous activators of aromatic hydrocarbon receptors [aryl hydrocarbon receptor (AhR)] [55]. So, the increment of BV in plasma of LB and HER2 cancer patients would suggest its implication in signaling and gene expression related to cell growth and cancer progression either by its increased plasma concentration, an up-regulation of the heme oxygenase-1 (HO-1) or a dysregulation of its catabolic enzyme biliverdin reductase (BLVR-A or BLVR-B) [56,57,58].

Moreover, not many studies have had an impact on our understanding on how the bile acid pattern differs in BC subtypes until now. Although an influence of bile acids on the development of breast cancer cells and the estrogen receptor function had been suggested [59], both pro and anti-proliferative effects of bile acids in different breast cancer cell models have been determined. Plasma deoxycholic acid (DA) concentrations were found to be higher in breast cancer patients than in controls without considering the BC molecular differences [60], while deoxycholate (DC) inhibited human luminal A breast cancer cell lines proliferation and glycochenodeoxycholate (GCDC) enhanced patient survival in another study [61]. In this aspect, our results show low levels of GUDCA in plasma of 21 luminal A cancer patients when compared with 21 healthy controls (FDR < 0.05 and FC < 0.06), which makes it interesting for further study in order to clarify its function in breast cancer development.

Finally, this study demonstrated that the four major BC subtypes could be discriminated using an untargeted metabolomics approach. Precise classification of these phenotypes has important implications in breast cancer personalized treatment, tailored follow up and detection of early recurrence.

## 4. Materials and Methods

### 4.1. Participants and Ethics

A total of 131 breast cancer patients and 134 healthy control subjects were recruited over 12 months at the Medical Oncology Unit of the University Hospital of Jaén (Spain). The study was approved by the Institutional Review Board of the Clinical Research Ethics Committee of Jaén and all clinical investigations were conducted under the Helsinki Declaration guidelines and the International Conference on Harmonization-Good Clinical Practices (ICH-GCP) guidelines. Every patient provided written informed consent for participation prior to blood sample extraction. The patient selection protocol was set as follows: female subjects being at least 18 years old with histological confirmation of BC, no detectable macro metastases and no previous anticancer treatment. Demographic details and clinical diagnosis of studied subjects are summarized in Table 4. The cancer stage was classified according to the 2002 Tumor Nodes Metastasis (TNM) system. Particularly, those BC patients diagnosed with HER2- and ER+ with Ki67 > 20% were defined as luminal B group and patients diagnosed with HER2- and ER+ with Ki67 < 20% were categorized as luminal A. As for non-luminal subtypes, all BC patients who neither expressed hormone receptors (PR-, ER-) nor overexpressed human epidermal growth factor 2 (HER2-) were considered as triple negative breast cancer patients; and finally, patients overexpressing human epidermal growth factor 2 were diagnosed as HER2+ breast cancer patients.

### 4.2. Plasma Sample Preparation

Samples were collected in EDTA tubes after at least 8 h fasting using standard venipuncture procedures. Blood was then centrifuged at 1400× *g* for 10 min at 4 °C and the supernatant was carefully aspirated and transferred into new vials, and immediately stored at −80 °C until the analysis.

### 4.3. Metabolite Extraction

An aliquot of 600 µL of acetonitrile (AcN) was added to 75 µL of plasma and the mixture was shaken for 2 min. Then, samples were centrifuged at 15,200× *g* for 10 min at 4 °C. The supernatant was collected in HPLC analytical vials. After that, it was evaporated in a GeneVac HT-8 evaporator (Savant, Holbrook, NY, USA) and kept frozen at −80 °C till the analysis. Finally, dry residues were reconstituted in 210 µL of water:AcN (50:50) with 0.1% formic acid and 250 ppb of L-leucine (1–13C, 99%), Roxithromycin, Caffeine-d3, Creatine (methyl-d3) monohydrate, L-abrine (methyl-d3) monohydrate and Bisphenol A-d16 as internal standards.

### 4.4. LC-HRMS Analysis

Samples were analyzed using an Agilent 1290 LC system (Agilent Technologies, Santa Clara, CA, USA) coupled to a quadrupole-time-of-flight 5600 mass spectrometer (AB SCIEX Q-TOF 5600, Concord, ON, Canada) in positive and negative electrospray ionization modes (ESI+, ESI−). A high performance liquid chromatography (HPLC) mode separation in ESI+ was carried out using an Atlantis T3 HPLC C18 column (2.1 mm × 150 mm, 3 µm; Waters Corporation, Milford, MA, USA) kept at 25 °C. Organic Solvent (OS) consisted of water:AcN (90:10) with 0.1% formic acid (eluent A) and AcN:water (90:10) with 0.1% formic acid (eluent B). The column was eluted with the following gradient: 0–0.5 min 1% eluent B; 0.5–11 min 99% eluent B; 11–15.50 min 99% eluent B; 15.50–15.60 min 1% eluent B and 15.60–20 min 1% eluent B. The elution flow rate was set at 300 µL/min [62]. Then, chromatographic separation was performed using a Gemini HPLC C18 column (100 mm × 2 mm, 3 µm; Phenomenex, CA, USA) kept at 25 °C in ESI− mode. The flow rate was 300 µL/min with mobile phases A (90% water: 10% AcN) and B (10% water: 90% AcN), both containing 0.1% ammonia at 20%. The gradient consisted of 0–0.3 min 1% eluent B; 0.3–7.3 min 99% eluent B, 7.3–10.3 min 99% eluent B and 10.3–13.3 min 1% eluent B. The TOF method operated with the Q-TOF 5600 allowed mass selection (80–1600 Da) with high resolution, in combination with an information dependent acquisition (IDA) method, which enabled the fragmentation of the eight most intense ions, to collect full-scan HRMS and MS/MS information simultaneously.

The exact mass calibration was automatically performed for every 10 injections of 5 µL of randomly injected plasma samples. Organic solvent samples were analyzed along the sequence for every 30 injections; quality control samples were analyzed for every 10 injections. The analysis of OS samples provided high impurity identification on either organic solvents or extraction procedure, and allowed discarding of carryover contamination. System stability and performance are evaluated by QC samples—a pool of equal volume of all plasma samples used in the study.

### 4.5. Data Processing

MarkerView software (version 1.2.1, AB SCIEX, Concord, ON, Canada) was used for LC-HRMS raw data processing. This tool performs peak detection, alignment and data filtering, providing a data matrix where the measured mass-to-charge ratio (*m/z*), retention time (RT) and intensities are defined for each sample. Afterwards, to minimize mass redundancy and enhance the true molecular feature selection, only monoisotopic peaks were considered. Background and contaminants were removed from the OS by applying an additional filtering procedure with fold change (< 1.5) and a *t* test (*p* > 0.05) between OS samples and study samples. Finally, according to FDA criteria for untargeted metabolomics, features with relative standard deviation higher than 30% were discarded because of their unacceptable variability in the QC samples [63]. The next steps were carried out using MetaboAnalyst 4.0 Web Server software (Wishart Research Group at the University of Alberta, Alberta, Canada) [64].

### 4.6. Normalization and Analytical Validation

Prior to the statistical analysis, normalization by a QC reference sample (probabilistic quotient normalization), transformation and scaling were applied to convert data set into a more Gaussian-type distribution [65,66]. Then, the PCA was used to assess the quality of the analytical system performance [67]. Analytical system stability was validated by QC samples presentation on a PCA plot. In parallel, the PLS-DA score plot showed possible outliers. Parameters R^2^ and Q^2^, which estimate goodness of fit and goodness of prediction respectively, were calculated to evaluate the statistical quality model description.

### 4.7. Statistical Analysis

Univariate analysis (UVA) was carried out using the non-parametric Wilcoxon rank-sum test to evaluate differences between BC patients and HC subjects. Benjamini-Hochberg false discovery rate (FDR) correction was performed afterwards to minimize the expected proportion of false positives (Type I errors) [68]. In this regard, a *p* value of 0.05 (corrected by FDR) for the *t* test is generally used in metabolomics as a cutoff threshold. Signals selected as potential candidates for a final discriminatory model were selected also based on their fold change (FC > 1.3). Eventually, a multivariate analysis was applied to identify features responsible for discriminating both study groups [30,69]. 

### 4.8. Metabolite Identification

PeakView software (version 1.0 with Formula Finder plug-in version 1.0, AB SCIEX, Concord, ON, Canada) was used to predict the elemental formula of selected candidates from accurate mass, isotopic clustering and fragmentation patterns. The assignment of a tentative identification for each selected metabolite was possible by searching different compound databases (Metlin, Human Metabolome Database, Lipid Maps, PubChem [70,71,72,73]) for accurate mass values. Structural identification of the molecular formula was achieved comparing the experimental fragmentation spectra against spectral databases (MassBank [74], NIST2014: version 2.2, Scientific Instrument Services, Inc, Ringoes, NJ, USA).

### 4.9. Biomarker Evaluation

Clinical relevance of the candidate metabolites was evaluated with the area under the receiver-operating characteristic curves (AUROC). In order to check the classifier performance of the biomarkers proposed for the diagnostic model, a multivariate ROC analysis was performed.

### 4.10. Pathway Analysis

MetaboAnalyst 4.0 Web Server software was used for the identification of altered metabolic pathways [64]. The metabolite ID matching was performed with Human Metabolome Database and KEGG database [71,75]. The analysis was adjusted by a hypergeometric test and the impact on pathway topology was based on relative-betweenness centrality.

## 5. Conclusions

Here we present an untargeted LC-HRMS metabolomics approach as a non-invasive technique to identify differential metabolomics signatures for BC subgroups. We found distinct molecular profiles representative for LA, LB, HER2 and TN BC phenotypes, which may act as crucial biomarkers for accurate diagnosis, phenotypic discrimination and personalized therapeutic intervention. It is worth highlighting the importance of a deep understanding of the molecular differences among BC subtypes within the realm of personalized medicine to avoid unnecessary side effects or inadequate target engagement. The metabolomics profiles discovered could be used as a powerful tool in clinical practice, not only to determine the existence of residual disease after neoadjuvant therapy and, thereby, contribute to the identification of patients who will absolutely benefit from additional treatment, but also to enlighten the development of new therapeutic strategies for each BC molecular subtype and tailored follow up. Finally, our findings reinforce a foundation to identify new biological targets in key metabolic pathways that may help to identify early subsequent relapses in the different BC phenotypes. Further analyses in larger prospective cohort of patients would be necessary to validate the prognostic/diagnostic capability of the different metabolomics profiles found among the four major BC subtypes.

## Figures and Tables

**Table 1:** Differential identified metabolites of molecular subtypes in BC.

BC Molecular Subtype	Tentative ID	*m/z*	RT	Mass Error (ppm)	*p* (FDR)	FC * (BC/HC)	Adduct	Molecular Formula
	**ESI+**							
**LB**	LysoPE(18:2)	478.2916	11.34	−2.5	1.670 × 10^−8^	0.6008	[M+H]	C23H44NO7P
	LysoPE(18:1(11Z/9Z))	480.3108	12.15	4.8	5.365 × 10^−3^	0.6303	[M+H]	C23H46NO7P
	LysoPE(18:1(11Z/9Z))	480.3073	12.47	−2.5	9.058 × 10^−10^	0.4713	[M+H]	C23H46NO7P
	LysoPC(20:3)	546.3539	12.16	−2.7	2.214 × 10^−2^	0.7303	[M+H]	C28H52NO7P
	Biliverdin	583.2566	8.95	2.6	7.390 × 10^−9^	1.5681	[M+H]	C33H34N4O6
**LA**	L-Tryptophan ^1^	188.0707	3.73	0.5	2.503 × 10^−2^	0.6362	[M+H-NH3]	C11H12N2O2
	LysoPC(14:0)	468.3084	9.66	−0.2	3.745 × 10^−2^	0.5849	[M+H]	C22H46NO7P
**HER2**	LysoPE(18:1(11Z)/9Z)	480.3109	12.31	5	6.192 × 10^−3^	0.6407	[M+H]	C23H46NO7P
	LysoPC(0:0/16:0)	496.3411	11.71	2.6	6.396 × 10^−6^	0.6701	[M+H]	C24H50NO7P
	Biliverdin	583.2525	8.65	−4.5	2.0621 × 10^−6^	1.6265579	[M+H]	C33H34N4O6
**TN**	L-Tryptophan ^1^	188.0702	3.4	2.1	4.153 × 10^−2^	0.625911	[M+H-NH3]	C11H12N2O2
	LysoPC(16:0/0:0)	518.3224	10.07	1.3	0.03043	0.5289669	[M+Na]	C24H50NO7P
**LB**	**ESI−**							
	LysoPE(16:0)	452.2796	5.71	2.9	5.427 × 10^−14^	0.5342	[M-H-H2O]	C21H44NO7P
	LysoPE(18:2)	476.2804	5.59	4.4	1.304 × 10^−8^	0.5498	[M-H]	C23H44NO7P
**LA**	L-Tryptophan ^2^	203.0824	1.27	−1	1.637 × 10^−2^	0.6543	[M-H]	C11H12N2O2
	Glycoursodeoxycholic acid ^3^	448.3066	3.24	−0.4	2.861 × 10^−2^	0.5646	[M-H]	C18H34O4
	LysoPE(18:2)	476.2766	5	−3.6	3.489 × 10^−2^	0.6711	[M-H]	C23H44NO7P
**HER2**	L-Tryptophan ^2^	203.0836	1	4.9	7.536 × 10^−5^	0.6744	[M-H]	C11H12N2O2
	LysoPE(18:2)	514.2381	5.5	7.8	3.403 × 10^−4^	0.6408	[M+K-2H]	C23H44NO7P
**TN**	LysoPE(18:1(11Z)/9Z)	957.5976	5.86	2.6	0.027908	0.4407772	[2M-H]	C23H46NO7P

Features statistically significant (FDR < 0.05 and FC > 1.3) with a tentative identification based on their accurate mass (*m/z*), MS/MS pattern or comparison with commercial standards ^1,2,3^, were selected to create the proposed multivariate model. * Fold change (FC) expressed as the ratio of two averages (BC/HC); BC varies depending on the molecular subtype. BC: breast cancer; HC: healthy control; LA: luminal A; LB: luminal B; HER2: overexpressing human epidermal growth factor 2; TN: triple negative.

**Table 2:** AUC scores of selected biomarkers (BM) for the proposed models and confusion matrices of the BC subtypes.

BC Molecular Subtype	BM	AUC	95% CI	Confusion Matrix	
				BC	HC
LA	5	0.87	0.651–0.992	14/20	16/21
HER2	5	0.919	0.819–0.985	26/31	28/34
TN	3	0.961	0.8–1	13/15	14/15
LB	7	0.954	0.886–0.995	50/56	54/62

**Table 3:** Altered pathways associated with BC molecular subtypes by ESI+ and ESI−.

Altered Pathways	BC Molecular Subtype	*p*-Value
Porphyrin and chlorophyll metabolism	LB and HER2	0.038347
Glycerophospholipid metabolism	LA, LB, TN and HER2	0.045927

**Table 4:** Demographic and clinical characteristics of breast cancer patients and healthy control subjects.

Characteristics	LB	HC	LA	HC	TN	HC	HER2	HC
**Subjects**	61	64	21	21	15	15	34	34
**Age (Range)**	49 (27–75)	50 (42–56)	50 (32–81)	49 (34–60)	49 (29–71)	51 (26–63)	51 (33–70)	49 (28–62)
**BMI (Kg·m^−2^)**	25.63 (16.9–40.5)	25.35 (19.8–30.0)	24.90 (20.0–37.2)	25.00 (18.0–28.3)	27.60 (21.60–41.23)	26.5 (21.3–30.0)	26.10 (21.0–33.3)	25.30 (20.80–29.80)
**HER2**	Negative	-	Negative	-	Negative	-	Positive	-
**PR**	Neg/Pos	-	Neg/Pos	-	Negative	-	Neg/Pos	-
**ER**	Positive	-	Positive	-	Negative	-	Neg/Pos	-
**Ki67**	>20%	-	<20%	-	-	-	-	-
**TNM-stage IA**	0	-	1	-	0	-	1	-
**TNM-stage IIA**	26	-	10	-	9	-	9	-
**TNM-stage IIIA**	12	-	0	-	0	-	3	-
**TNM-stage IIB**	19	-	9	-	3	-	19	-
**TNM-stage IIIB**	2	-	1	-	2	-	1	-
**TNM-stage IC**	2	-	0	-	1	-	1	-

HC and BC patients were matched in terms of age and BMI. BC: breast cancer; LB: luminal B; HC: healthy control; LA: luminal A; TN: triple negative; HER2: human epidermal growth factor receptor 2 positive; BMI: body mass index; PR: progesterone receptor; ER: estrogen receptor; TNM: tumor nodes metastasis.

## Data Availability

All data generated or analyzed during this study are included in this published article and its supplementary materials. Raw data are not publicly available due to ethical restrictions, since they contain information that could compromise the privacy of research participants, but are available from the corresponding author on reasonable request.

## Supplementary Materials

The following are available online at https://www.mdpi.com/2072-6694/13/1/147/s1. Figure S1: 2D score plots of the unsupervised PCA of HC group (green) and LA_BC (light blue) (**a**), HER2_BC (orange) (**b**), TN_BC (yellow) (**c**) and LB_BC (pink) (**d**) patients by ESI+ showed that the separation observed between the groups was due to biological reasons according to the close clustering of the QC samples (dark blue). Figure S2: 2D score plots of the supervised PLS-DA of HC group (green) and LA_BC (light blue) (**a**), HER2_BC (orange) (**b**), TN_BC (yellow) (**c**) and LB_BC (pink) (**d**) patients by ESI− determined a notably separation between BC molecular subtypes and matched controls. Figure S3: Characteristic MS/MS spectra of *m/z* 448.3066 in a biological sample (green) (**a**) and the glycoursodeoxycholic acid (GUDCA) standard (blue) (**b**) at 3.24 min. MS/MS spectra revealed the characteristic fragmentation pattern of GUDCA in ESI−. **Table S1**: Extracted peaks from RPLC ESI+ and ESI− HRMS, significant altered metabolites and quality model description. Table S2: Features identified by accurate mass (*m/z*) and retention time (RT). Table S3: Altered non-significant pathways associated with BC molecular subtypes by ESI+ and ESI−.
