# Supplementary material for: Human Plasma Metabolomics for Biomarker Discovery: Targeting the Molecular Subtypes in Breast Cancer
Source: Cancers (Basel). 2021 Jan 5;13(1):147. doi: 10.3390/cancers13010147 (PMC7795819; doi:10.3390/cancers13010147)
Supplement: Supplementary file 1 [file cancers-13-00147-s001.pdf]

cancers MDPI

Supplementary materials: Human plasma metabolomics for biomarker discovery: Targeting the Molecular Subtypes in Breast Cancer

Leticia Díaz-Beltrán, Carmen González-Olmedo, Natalia Luque-Caro, Caridad Díaz, Ariadna Martín-Blázquez, Mónica Fernández-Navarro, Ana Laura Ortega-Granados, Fernando Gálvez-Montosa, Francisca Vicente, José Pérez del Palacio and Pedro Sánchez-Rovira

Table S1. Extracted peaks from RPLC ESI+ and ESI- HRMS, significant altered metabolites and quality model description.

BC molecular Subtype	Total	Monoisotopics	RSD>30%	OS filtering	PCA evaluation	Altered metabolites	R^2	Q^2
				ESI +				
**LB**	1497	542	168	255	119	80	0.76	0.6
**LA**	1322	419	99	152	168	62	0.75	0.49
**TN**	2129	529	261	93	175	7	0.95	0.38
**HER2**	2384	646	108	344	194	115	0.84	0.55
				ESI -				
**LB**	1584	410	8	154	248	197	0.99	0.98
**LA**	821	144	17	77	50	32	0.98	0.96
**TN**	1254	309	5	52	252	10	0.96	0.59
**HER2**	1467	442	5	80	357	164	0.7	0.45

Marker View software provided a data matrix containing the extracted peaks. This software allowed us to apply different filter steps to finally detect the features responsible for the discrimination between the groups. The non-parametric Wilcoxon rank-sum test (*p*, FDR< 0.05) was used to validate the significance of the difference in intensities between variables. R^2 and Q^2 parameters were calculated to evaluate the statistical quality model description.

Table S2. Features identified by accurate mass (m/z) and retention time (RT).

BC molecular Subtype	m/z	RT	*p* (FDR)	FC (BC/HC)*
			ESI+	
LB	188.0694	3.54	0.000181	0.661659764
	424.3436	9.88	0.047649	0.720931714
	494.3241	10.94	0.016454	0.696788467
	500.2729	11.35	1.01E-09	0.567782517
	502.2877	12.43	2.93E-09	0.526618524
	508.3396	11.77	0.047049	0.765045405
	516.3074	10.96	0.036772	0.702130928
	518.3239	10.68	0.00633	0.649721236
	520.3401	11.53	5.43E-07	0.632905958
	522.3486	11.44	4.61E-05	0.62972251
	522.3588	12.72	0.022614	0.719381574
	539.3099	11.51	2.70E-08	0.548812571
	541.3302	12.7	7.17E-06	0.605368254
	544.3354	12.61	0.044136	0.707257547
	544.3392	12.74	0.027313	0.730365454
	548.3685	13.28	0.049492	0.747556702
	558.2988	11.52	0.001147	0.702232228
	566.3205	11.46	0.031742	0.721545192

	762.981	12.2	0.001961	0.719055992
	798.9749	11.47	3.73E-09	0.420999989
	801.9816	11.53	3.36E-09	0.447644268
	991.6758	12.19	0.000488	0.681636976
	1002.6569	12.22	8.65E-05	0.651087195
	1010.6433	12.21	3.75E-05	0.660973934
	1014.157	12.17	0.00117	0.688398189
	1017.6837	12.31	2.45E-07	0.603190274
	1041.69	12.19	1.71E-06	0.610901465
	1043.6957	12.72	9.83E-07	0.493623718
	1061.6563	11.51	3.73E-09	0.426939569
	1062.6515	11.52	3.10E-09	0.41806545
	1063.6633	11.5	2.53E-09	0.45355252
	1065.689	12.7	1.56E-07	0.477682583
	1085.6556	11.51	3.31E-07	0.533432221
	1261.3227	12.19	0.000185	0.607528629
	1508.9915	12.22	0.000198	0.613783086
**LA**	482.3212	10.63	0.008477	0.612964935
	518.6578	10.86	0.034272	0.635888274
	521.3444	10.88	0.039792	0.638710336
	522.3439	10.61	0.011563	0.59654187
	542.3196	10.61	0.037448	0.604686277
	758.4347	10.61	0.02503	0.605662817
	798.9807	10.86	0.016881	0.457333326
	801.982	10.86	0.032804	0.541499037
	810.978	10.87	0.008477	0.510034942
	991.6718	11.4	0.005927	0.607791282
	997.6209	10.81	0.014809	0.451747844
	1002.659	11.4	0.014809	0.658313957
	1010.643	11.41	0.014809	0.646775446
	1062.65	10.85	0.007173	0.472831286
	1063.661	10.87	0.003475	0.412957109
	1085.646	10.88	0.011563	0.573309571
**HER2**	329.2457	15.87	0.001163	1.329118996
	450.3221	9.66	0.015067	0.573750651
	476.2761	10.45	0.014926	0.68770148
	480.3111	12.01	0.043154	0.67273135
	496.3396	12.06	0.019999	0.712534412
	502.2883	12.31	0.033225	0.683612
	515.3145	12.05	0.019433	0.727539976
	515.2627	4.15	0.002221	0.374716794
	521.3429	11.31	0.017469	0.689239322
	522.3458	11.11	0.02931	0.701052267
	524.3716	13.97	0.039263	0.706184276
	542.3228	11.31	0.011049	0.639305036
	542.3228	11.31	0.011049	0.639305036
	585.2708	7.3	7.34E-05	1.527313773

	599.4391	12.14	0.000144	0.658826728
	610.3079	11.11	0.007454	0.68190286
	623.4416	11.38	0.048891	0.661109942
	754.992	12.09	3.65E-07	0.615305186
	762.9782	12.09	3.66E-06	0.617498696
	765.9881	12.05	0.00019	0.695053185
	771.9705	12.05	0.002302	0.650196234
	798.9802	11.37	0.011482	0.591346447
	801.9857	11.41	0.014926	0.642295525
	805.0046	12.57	0.003006	0.615981099
	810.9778	11.39	0.003427	0.621959193
	932.5261	11.72	2.56E-05	0.62900932
	955.5781	11.23	0.00458	0.626984118
	956.5262	11.12	0.007454	0.558480779
	991.6731	11.72	4.66E-08	0.350103215
	991.6721	12.06	8.50E-08	0.512209654
	1002.661	12.05	4.66E-08	0.508449895
	1010.637	12.06	1.21E-07	0.519220446
	1013.6497	11.71	8.50E-08	0.312418306
	1013.6568	12.08	5.74E-05	0.689294135
	1014.1575	12.06	2.25E-07	0.583683555
	1017.6787	12.24	2.53E-05	0.564801879
	1039.6682	11.37	0.003546	0.522726991
	1039.6638	12.19	8.01E-05	0.63472339
	1041.7014	12.02	0.000903	0.605781811
	1043.6986	12.59	0.012336	0.629198894
	1061.6562	11.37	0.011482	0.642010974
	1063.6685	11.39	0.002633	0.574875198
	1065.667	11.37	0.005994	0.601601277
	1065.6847	12.59	0.010275	0.627315539
	1073.6651	11.38	0.00458	0.589103205
	1085.6489	11.39	0.044253	0.694602855
	1258.3102	12.08	6.52E-07	0.447635622
	1258.81	12.07	6.52E-07	0.427070417
	1261.3215	12.05	4.66E-08	0.442607045
	1261.8226	12.05	2.25E-07	0.458332604
	1508.9825	12.08	2.25E-07	0.445220732
**TN**	409.1587	9.31	0.03043	1.807452654
	425.1359	9.33	0.03043	1.820459243
			**ESI -**	
**LB**	199.1707	3.72	1.45E-11	1.438373242
	225.1863	3.93	6.11E-11	1.443604784
	238.0805	2.32	1.05E-05	1.582350023
	241.1963	5.6	6.49E-16	1.310097924
	303.2349	4.73	1.53E-14	1.596269769
	305.2499	4.87	2.43E-10	1.388192357
	331.1923	4.53	3.17E-16	2.292562288

355.1701	6.64	5.46E-20	3.17342607
367.1612	3.32	1.35E-06	1.649502118
369.1765	3.11	9.42E-06	1.40701092
369.1744	3.54	5.75E-08	1.824429281
383.1538	2.9	1.96E-07	1.616166787
385.1716	2.64	6.24E-08	1.696586057
385.1812	6.64	5.46E-20	3.108817515
391.289	3.52	0.000788	0.317178793
395.1914	3.71	2.88E-11	2.197940531
397.2086	3.51	1.70E-10	2.168436878
397.206	3.87	0.000182	2.005603465
399.2234	3.6	9.02E-07	2.615232098
407.2795	3.27	0.000429	0.203073672
409.2392	7.56	6.15E-07	0.661343969
413.2028	3.21	1.45E-11	1.720133144
421.1571	6.64	5.46E-20	2.96387748
427.2173	3.54	8.52E-05	1.426932244
435.2545	7.57	3.40E-07	0.674320154
435.2536	7.8	0.000462	0.688626077
436.2862	5.89	3.82E-09	1.395241773
437.2716	8.46	1.22E-15	0.439588975
437.2714	8.69	5.55E-07	0.563739994
448.1777	6.64	5.46E-20	2.920654647
449.1532	3.73	5.46E-20	12.42037049
459.2552	7.19	8.99E-06	0.637042437
460.2592	7.2	1.31E-05	0.630757381
461.2718	7.86	9.46E-05	0.675847406
462.3005	6.13	1.03E-12	1.637399978
465.2536	3.2	5.58E-06	1.514013551
465.3061	6.45	1.72E-12	1.522051505
473.3672	4.04	0.040611	0.603563783
478.2981	5.92	6.27E-14	0.409165151
479.3729	4.12	2.98E-14	1.45323045
480.3056	6.43	0.000224	0.605032068
494.323	7.56	1.49E-06	0.663194812
495.3005	3.44	0.002154	2.071991354
498.2624	5.53	6.95E-07	0.586808354
500.2796	5.92	5.31E-12	0.446895021
508.2844	5.68	5.46E-20	2.256078377
511.2949	3.22	0.000237	1.313327037
518.3237	7.18	2.88E-06	0.626038938
537.3326	7.57	0.002128	0.699994088
540.2727	4.05	1.63E-14	0.466732668
540.2757	4.59	5.46E-20	0.047582144
540.3368	7.48	4.63E-06	0.630137922
540.3315	7.56	6.82E-07	0.66062606
540.2727	9.88	5.46E-20	0.186376564
540.8199	1.66	1.05E-09	1.361998162
554.3434	7.56	2.11E-05	0.672001144
556.3287	7.55	2.85E-08	0.660066798
557.4643	4.55	6.33E-10	0.341584753

558.3401	7.48	3.67E-05	0.655130023
560.2705	5.53	6.09E-06	0.641304003
561.3341	7.19	0.000197	0.650665458
563.3499	7.81	0.046753	0.718724427
564.337	7.17	6.09E-06	0.631363624
566.3444	7.81	0.001027	0.695284278
567.5368	5.24	2.19E-12	1.353710489
568.3603	8.45	1.92E-15	0.434058749
568.3675	8.69	1.68E-07	0.516084703
570.3444	7.97	0.003009	0.653275679
573.4619	4.08	0.007093	0.515972634
573.4588	4.24	0.002642	0.487966171
578.3528	7.17	4.74E-06	0.628832325
580.3332	7.17	4.82E-06	0.624295471
580.371	7.82	0.000207	0.672308285
582.3374	7.17	7.42E-05	0.651869084
582.3459	7.8	0.000392	0.686786579
584.3583	8.45	3.06E-15	0.441814117
584.3656	8.67	2.97E-07	0.549971899
585.361	8.65	7.02E-07	0.552641203
590.3195	7.5	0.000207	0.659589983
590.3223	7.56	8.39E-05	0.698952048
591.4696	3.94	0.027099	0.479219449
593.4829	4.13	0.017147	0.585140289
602.7984	1.64	2.22E-15	1.337511598
606.3402	8.49	8.01E-15	0.469353112
608.3642	8.09	7.02E-07	0.640526956
614.3166	7.13	5.10E-05	0.656174146
616.3321	7.82	0.002674	0.705227251
619.2935	4.72	1.92E-07	0.570427603
659.4169	8.49	0.000423	0.668615024
668.3394	8.45	9.26E-15	0.46417904
668.34	8.68	0.003998	0.666116978
671.2656	9.88	9.38E-06	0.586439383
678.3005	9.88	1.20E-09	0.51610498
681.3031	9.88	1.93E-10	0.506723816
695.3179	9.87	8.10E-10	0.508550763
697.298	9.88	6.47E-11	0.495852767
698.4262	11.22	5.46E-20	2.459756411
712.4004	10.93	1.70E-10	0.398465484
714.4221	10.11	5.46E-20	5.578054658
714.417	10.91	6.81E-19	1.748179883
714.4107	11.25	1.31E-13	0.487365074
730.415	11.12	5.46E-20	0.110698181
730.4133	9.35	5.46E-20	3.088994098
730.4138	9.36	0.043704	0.725181709
730.4107	9.9	1.48E-19	2.159996657
734.4027	11.22	5.46E-20	2.3543829
746.4014	10.9	5.46E-20	0.032685023
746.4076	9.88	1.50E-14	0.398782137
750.3965	10.09	5.46E-20	4.526014424

	750.3987	11.29	4.63E-12	0.472602827
	757.4203	11.28	6.97E-08	0.5582306
	761.4241	11.23	5.46E-20	2.020445815
	764.3618	10.95	1.46E-17	0.087260903
	766.394	11.11	5.46E-20	0.130556068
	766.382	9.39	3.22E-16	0.397493252
	768.3951	9.36	8.21E-16	0.386022448
	775.4007	10.9	7.91E-11	0.413058921
	777.4156	10.09	5.46E-20	3.577938579
	777.4163	11.32	1.00E-13	0.457894786
	782.3805	10.86	5.46E-20	0.035440369
	791.3928	10.98	8.25E-18	0.090092625
	793.4098	11.04	5.46E-20	0.129887089
	904.5777	7.55	1.14E-12	0.546884212
	918.5875	7.55	2.06E-12	0.531768963
	952.5745	7.18	3.17E-09	0.481593909
	956.6125	7.84	3.19E-08	0.552639615
	976.5738	7.24	1.24E-05	0.598945011
	989.6758	7.56	2.01E-13	0.54914282
	1035.6755	7.56	6.92E-10	0.551998883
	1037.6753	7.19	9.40E-09	0.493304993
	1041.6998	7.83	1.70E-08	0.538034313
	1051.6721	7.46	1.22E-15	0.353525035
	1051.6559	7.56	5.69E-13	0.552133485
	1073.6529	7.53	0.00057	0.695610816
	1099.6742	7.17	6.75E-09	0.488465793
	1103.698	7.82	1.37E-08	0.532730131
	1121.657	7.17	8.38E-07	0.586531839
	1123.6707	7.2	2.36E-05	0.60350591
	1135.6532	7.55	0.002642	0.70277207
	1183.6658	7.17	4.74E-06	0.592968652
	253.2182	4.03	0.01134	1.543582497
	255.2339	4.26	3.72E-06	1.395733146
	281.2487	4.33	0.030446	1.88602715
	286.214	4.27	7.17E-06	1.500205102
	313.2395	4.36	0.001924	1.97821063
	351.2194	4.29	4.71E-06	1.380483283
	367.1581	3.13	0.038789	0.629369591
	377.2347	4.37	0.031318	1.869997431
**LA**	389.2745	4.37	0.030446	1.831135114
	391.2821	3.49	0.025021	0.233415595
	407.2761	3.25	0.046014	0.134123139
	413.1983	5.99	6.19E-11	3.122262519
	447.1301	3.56	3.60E-09	0.30481595
	465.3038	5.12	6.19E-11	5.101262081
	500.2743	4.98	0.005684	0.693522754
	523.2927	4.07	0.034844	1.410911548
	524.2733	4.98	0.018667	0.732511821
	540.272	4.02	6.19E-11	0.02640082

	551.3252	4.36	0.031318	1.727496137
	580.3218	11.84	0.018667	0.577213127
	602.3004	11.82	0.005586	0.582257098
	613.2726	4.07	0.020789	1.44768918
	641.3003	4.38	0.02779	1.791668744
	643.317	4.6	8.42E-09	2.052066351
	653.2979	4.49	5.57E-10	0.157001633
	664.301	11.84	0.002697	0.560615407
	225.1866	3.89	0.002148	1.409120314
	253.2174	4.36	0.002084	1.366445531
	277.2179	4.3	0.041601	1.560883434
	279.2338	4.53	0.004836	1.555074692
	281.2475	4.83	0.009389	1.473024803
	297.2451	4.83	0.004429	1.54607755
	303.2338	4.61	0.003754	1.428186991
	309.2819	5.26	0.006765	1.307058039
	377.236	4.83	0.006123	1.467860128
	409.2356	7.36	0.001598	0.747175491
	416.8413	1.89	9.28E-08	0.638046131
	448.3069	3.57	0.047398	0.747279385
	450.8298	1.94	2.17E-05	0.708987921
	465.8403	1.97	2.38E-08	1.534379444
	480.3101	7.49	0.031827	0.759247047
	486.2645	3.57	0.002587	0.603816223
	494.3294	7.36	0.000761	0.747991165
	508.3434	8.41	0.020992	0.765569101
	508.3426	8.61	0.017295	0.763137463
	522.804	2.09	4.77E-07	0.628761733
**HER2**	538.7805	2.11	9.27E-09	0.420443774
	540.3342	7.34	0.005021	0.756415884
	550.7944	2	1.98E-06	0.671107518
	563.5014	4.83	0.014832	2.427858684
	568.3643	8.42	0.021481	0.752554061
	572.7695	2	5.94E-08	0.549397622
	583.7535	2.11	8.14E-08	0.514337475
	584.3601	8.41	0.014095	0.739897009
	584.3567	8.61	0.024108	0.763099038
	593.8019	1.89	2.60E-08	1.356101965
	611.4896	4.82	0.021481	1.772913291
	611.7626	1.94	4.71E-06	0.663956408
	628.7725	1.84	5.31E-05	0.708331848
	643.777	2.04	3.65E-08	1.305538691
	644.7408	2	1.36E-08	0.461977904
	660.7137	2	1.36E-08	0.440777209
	676.694	2.06	1.36E-08	0.428084929
	678.7327	1.94	3.65E-08	0.586403516
	694.7091	2	9.28E-08	0.553390049
	714.7602	1.94	0.000928	0.72510449
	736.7468	2.01	0.002905	0.730976843

	742.747	1.93	0.025722	0.759168768
	750.7213	2.09	6.29E-08	0.605366658
	751.7348	1.84	0.002795	0.703461577
	768.7278	1.93	0.005619	0.745626815
	771.7401	1.89	1.05E-08	1.634477131
	778.6954	1.96	1.65E-07	0.588741679
	784.6968	2.08	3.05E-07	0.644847895
	790.701	1.94	6.26E-05	0.699017898
	796.6584	1.92	0.041601	0.765151292
	817.6818	1.94	9.28E-08	0.523775149
	856.6782	1.93	1.72E-06	0.655711746
	862.6614	1.93	5.83E-07	0.625372605
	872.642	2.11	1.74E-07	0.610930585
	882.699	2	0.046318	0.766298791
	904.5728	7.34	3.34E-05	0.631142893
	904.5692	7.53	0.008125	0.74504555
	918.5862	7.53	0.006592	0.740383453
	952.5661	7.16	0.022024	0.716582334
	952.6562	2	0.001047	0.670422214
	953.5715	5.48	0.030844	0.720157173
	960.6318	8.61	0.006765	0.658813078
	978.6174	2.04	6.29E-08	0.603800853
	989.6597	7.53	8.29E-05	0.735065347
	1035.661	7.53	0.001757	0.7445087
	1037.67	7.16	0.014458	0.700021507
	1051.663	7.34	5.56E-05	0.62213411
	1051.658	7.54	0.000394	0.749866322
	1061.651	7.17	0.038037	0.738628266
	1099.674	7.18	0.012703	0.702771133
	1107.727	8.63	0.000539	0.620406573
	1546.993	7.53	0.002005	0.669029124
**TN**	824.7259	1.29	0.001651	2.006859929

Potential biomarkers were selected according to the non-parametric Wilcoxon rank-sum test (*p*, FDR< 0.05) and fold change > 1.3. Identification of these features was based on their accurate mass (m/z) and retention time since their tentative ID could not be clarify by comparison of MS/MS spectra or commercial standards. *Fold change (FC) expressed as the ratio of two averages (BC/HC); BC varies depending on the molecular subtype. LA: luminal A; LB: luminal B; HER2: overexpressing human epidermal growth factor 2; TN: triple negative.

Table S3. Altered non-significant pathways associated with BC molecular subtypes by ESI + and ESI -.

**Altered pathways**	**BC molecular subtype**	***p* Value**
Tryptophan metabolism	LA, HER2 and TN	> 0.05
Aminoacyl-tRNA biosynthesis	LA. HER2 and TN	> 0.05

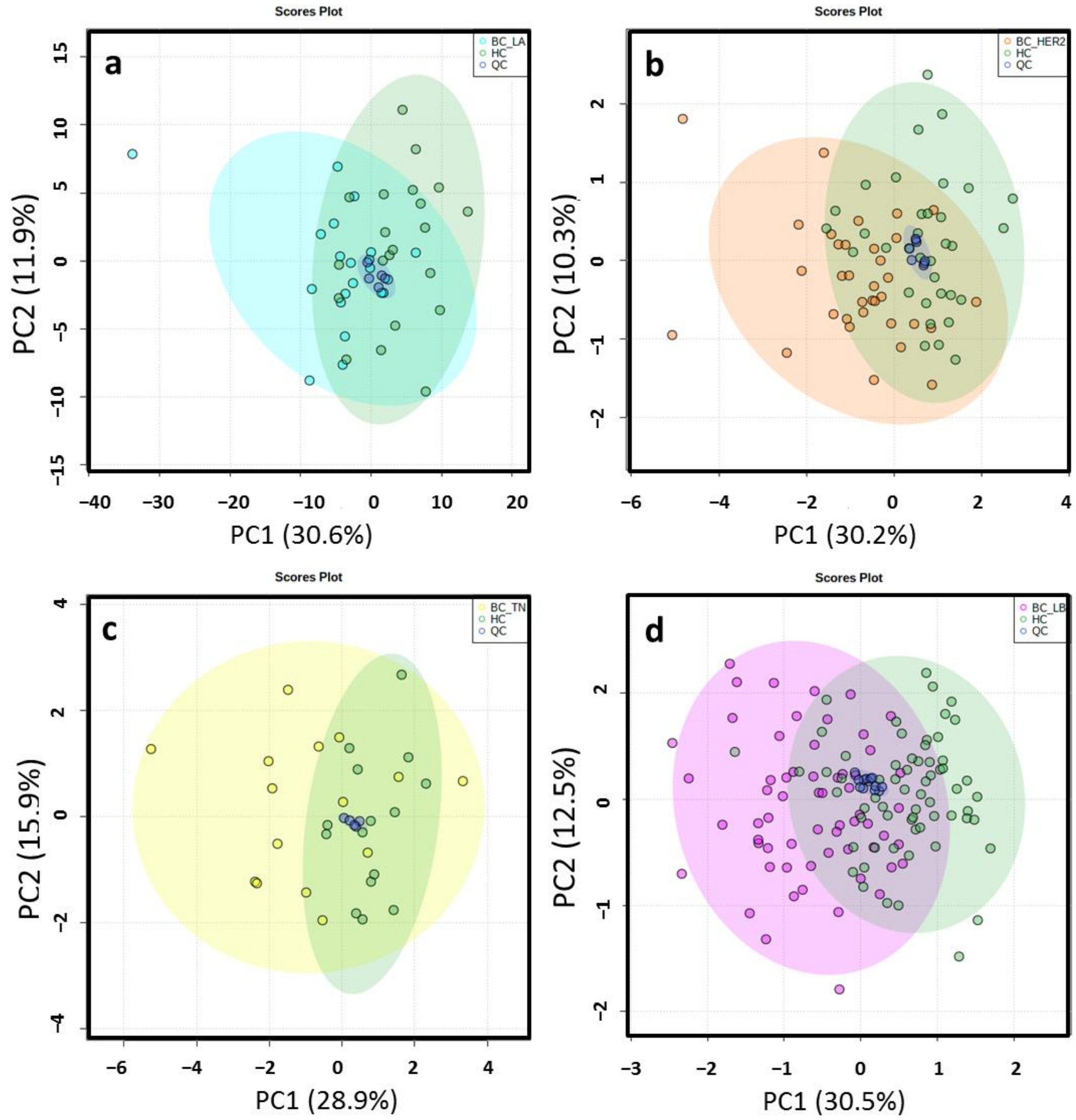

Figure S1. 2D score plots of the unsupervised PCA of HC group (green) and LA_BC (light blue) (**a**), HER2_BC (orange) (**b**), TN_BC (yellow) (**c**) and LB_BC (pink) (**d**) patients by ESI + showed that the separation observed between the groups was due to biological reasons according to the close clustering of the QC samples (dark blue).

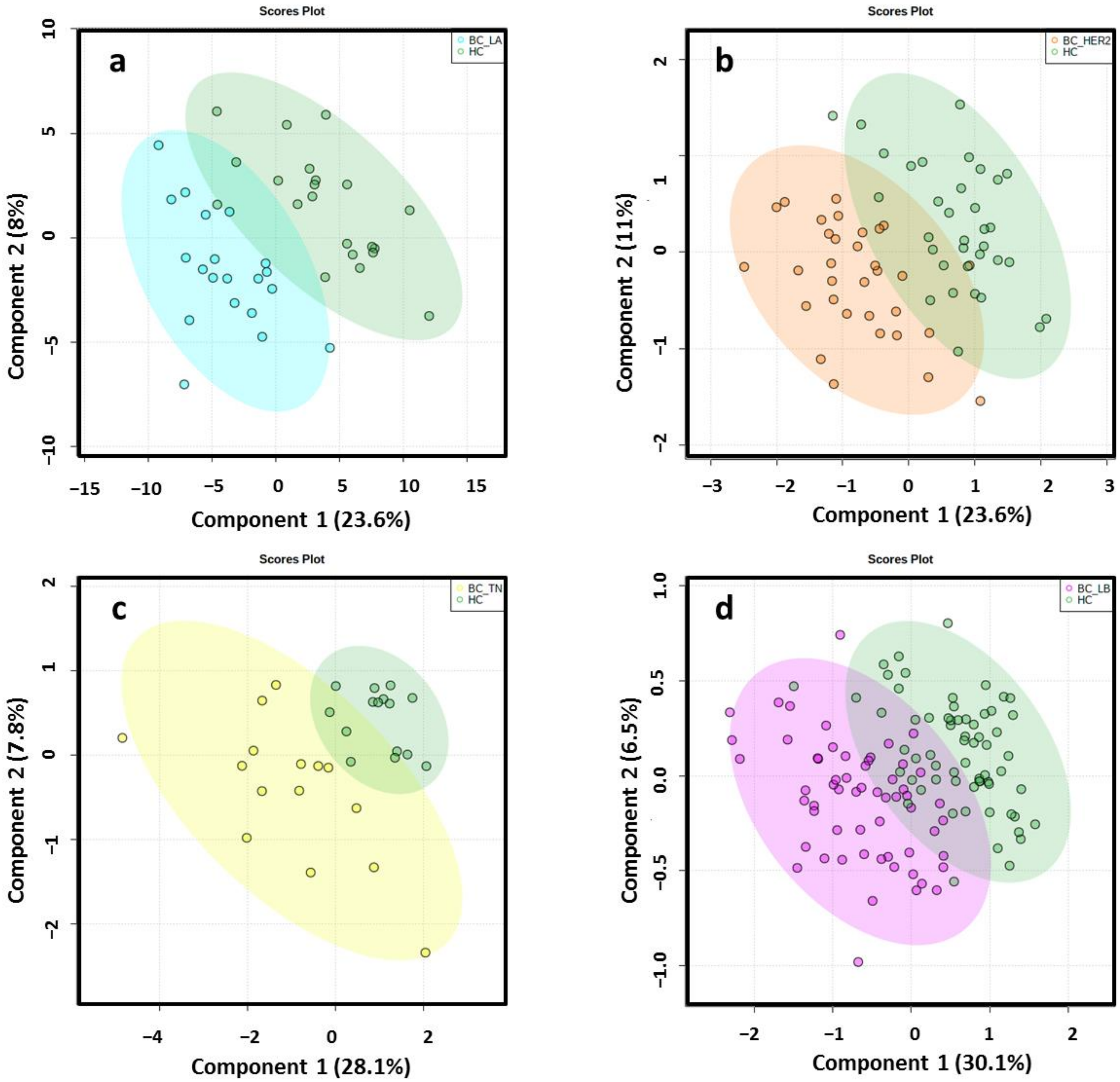

Figure S2. 2D score plots of the supervised PLS-DA of HC group (green) and LA_BC (light blue) (**a**), HER2_BC (orange) (**b**), TN_BC (yellow) (**c**) and LB_BC (pink) (**d**) patients by ESI+ determined a notably separation between BC molecular subtypes and matched controls.

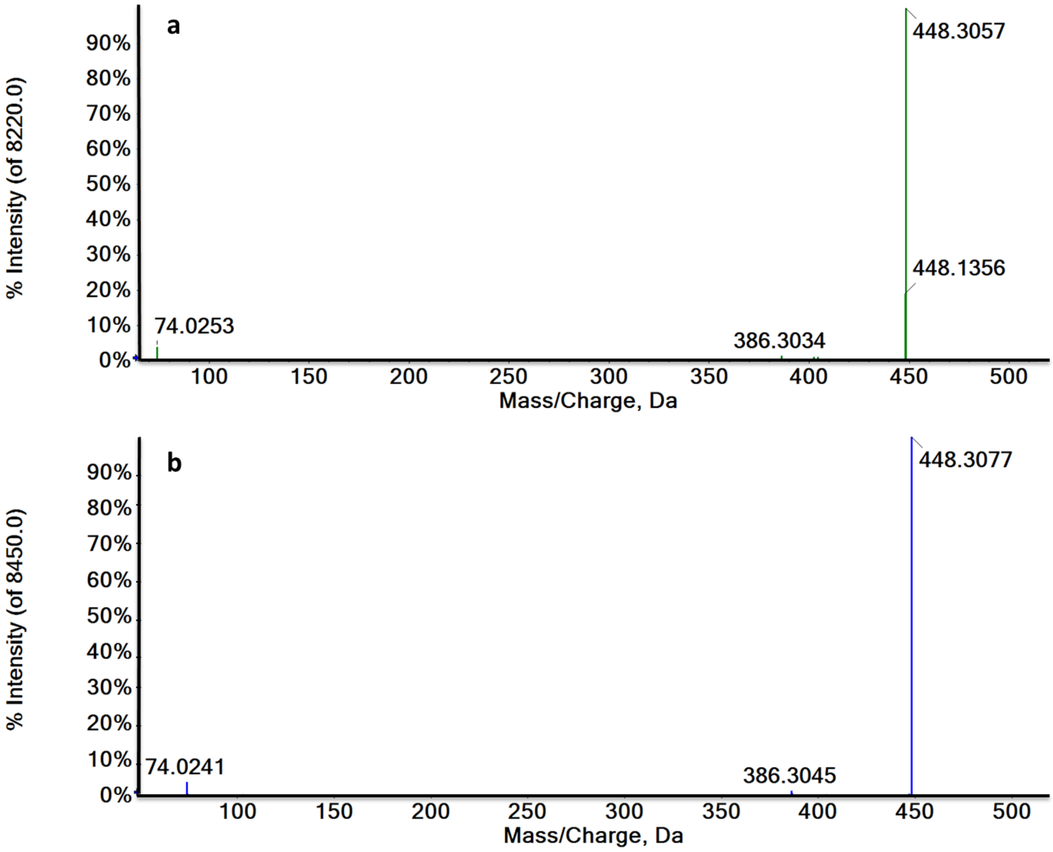

Figure S3. Characteristic MS/MS spectra of m/z 448.3066 in a biological sample (green) (**a**) and the glycoursodeoxycholic acid (GUDCA) standard (blue) (**b**) at 3.24 min. MS/MS spectra revealed the characteristic fragmentation pattern of GUDCA in ESI-.
